# Editorial Comment: Upper urinary tract stone compositions: the role of age and gender

**DOI:** 10.1590/S1677-5538.IBJU.2019.0278.1

**Published:** 2020-01-13

**Authors:** Fábio C. M. Torricelli

**Affiliations:** 1 Divisão de Urologia, Hospital das Clínicas, Faculdade de Medicina da Universidade de São Paulo. São Paulo, SP, Brasil

Kidney stones prevalence is increasing worldwide, leading to a higher number of patients requiring medical treatment (clinical or surgical approaches) (1, 2). It has important impact on social and economic life of people suffering from urinary stones, bringing significant financial costs and becoming a relevant problem to healthcare managers. Understanding kidney stones etiology (and composition), its specific risk factors and how to prevent its formation, growth, and complications is essential to a good medical practice.

In this retrospective study including more than 1,500 stone analysis, authors have shown the influence of gender and age on stone composition. Men presented with more CaOx and UA stones, whereas the prevalence of infection and CaP stones was higher in women (3). Regarding age, prevalence of UA stones increased with aging while prevalence of infection stones decreased in both genders. Lieske et al. in a study with 43,545 stone analysis have already linked gender and age to stone composition. While women have presented with a higher prevalence of hydroxyapatite and struvite stones, men have presented with a higher prevalence of calcium oxalate and uric acid stones. Aging was also associated with a higher prevalence of uric acid stones (4). Others two studies, one from Israel and another from Turkey have also found similar outcomes (5, 6). In all studies CaOx is the most common kidney stone, regardless of age and gender, however some patterns on stone composition distribution can be noted according to gender and age.

It is exciting to know that stone composition may be associated with age and gender, but we have to be careful when interpreting these data because others factors such as dietary modifications, BMI changes over time and postmenopausal alterations in women may play also important role on stone composition.
